# Examining the COVID-19 pandemic and its impact on social work in health care

**DOI:** 10.1177/14680173221142767

**Published:** 2023-01-03

**Authors:** David B. Nicholas, Patricia Samson, Leeann Hilsen, Janet McFarlane

**Affiliations:** Faculty of Social Work, 2129 University of Calgary, Central and Northern Alberta Region, Edmonton, Alberta, Canada; 3146 Alberta Health Services, Calgary, Alberta, Canada

**Keywords:** Social work, health care, COVID-19, practice, program shifts, impacts

## Abstract

**Summary:**

This qualitative study examined the COVID-19 pandemic as experienced by healthcare-based social workers in relation to practice, and personal and professional impacts of providing care in this context, with recommendations for pandemic preparedness and response. A total of 12 focus groups were convened between June 2020 and March 2021, comprising 67 hospital social workers across multiple hospitals and other care facilities in western Canada.

**Findings:**

Based on an Interpretive Description approach, themes emerged reflecting practice shifts; increased work and changing roles; imposed restrictions; problems in communication and decision-making; distress, fear, and demoralization; and co-existing *silver linings* amid challenges.

**Applications:**

The COVID-19 pandemic has substantially impacted social workers and their delivery of service. Addressing concerns through proactive responsiveness, both during and beyond the pandemic, are important in nurturing patient-centered care and a supported workforce. Along with that of interdisciplinary colleagues in health care, social workers’ practice has been profoundly impacted by the COVID-19 pandemic. This article explores the experiences of social workers in healthcare settings during the COVID-19 pandemic.

## Introduction

The experiences and impacts of the COVID-19 pandemic on healthcare-based social workers are not yet well-documented in the literature. A greater understanding about social workers’ roles and experiences in such circumstances is important in determining this experience and providing guidance for social work as a profession in the potential event of a pandemic or other health crisis. This study elicited the perspectives of social workers in health care regarding the impacts of the COVID-19 pandemic on their experience and practice.

## Background

As noted by Cheung (2020), “[t]he role of social work in pandemics has long been significant throughout the years but has not been well documented in literature” (p. 213). Gearing et al. (2007) studied the experiences of hospital-based social workers during the 2003 SARS pandemic in Toronto, Canada. Social workers in that sample reported needing to manage the heightened emotions of patients and families; struggling with internal emotions themselves; needing to build their own support systems and coping strategies; facing challenges with technology and effective communication, especially at the onset of the pandemic; experiencing challenges related to communication due to personal protective equipment (PPE) (e.g., mask use) as well as visitation and isolation protocols; and engaging in advocacy and bridging for patients, families, other professionals, often in fluctuating policies. Social workers were described to require a great deal of flexibility and adaptability to carry out their roles in the hospital environment (Gearing et al., 2007).

The ubiquitous nature of COVID-19 has meant that social workers have navigated struggles, concerns, and fears similar to those of their clients, negatively impacting personal boundaries and coping (Ross et al., 2021; Wilson et al., 2021). It has been noted that social workers have felt significant stress from the challenge to meet clients’ COVID-related and other health needs, with limited resources and restricted contact (Wiener et al., 2021). They were reported to have experienced perceived or actual pressure to do more to meet increased demand, along with feelings of inadequacy in not doing enough (Ross et al., 2021). Wiener et al. (2021) noted that “the existing inequities in access to critical resources and emotional support (caused) social workers to experience moral distress associated with delivering psychosocial care…” (p. 430).

Banks et al. (2020) noted a number of ethical challenges that have arisen for social workers during the pandemic: needing to create and maintain relationships in an environment requiring social distancing and PPE, which could impede communication (such as limiting nonverbal communication and touch); navigating increased service demands in an environment of service cuts; managing risks of disease transmission versus providing face-to-face services; potentially breaching government and agency policies in order to provide needed services; and taking care of oneself while working in potentially unsafe and stressful situations. Abrams and Detlaff (2020) conveyed how social workers have made ethical decisions moment-to-moment, citing an example of a practitioner weighing the risks and benefits of wearing or not wearing PPE to avoid frightening a young client. Xenakis et al. (2021) described the redistribution of social workers outside of their specialities, and the required assumption of increased administrative and coordination roles related to patient and family care.

Booth and Venville (2020) identified the unintended consequences of COVID-19 response measures from the perspective of hospital social workers in Melbourne, Australia. At the forefront has been the all-encompassing impact of COVID-19 on practice, services, and patient care. Specifically noted were the challenges of PPE on communication and connection; logistical scheduling of business hours and visiting hours, thus creating a disconnect between social workers and families; increased distress, isolation, and conflict for patients due to visitor restrictions; ward-based practice rather than practice within one's speciality; isolation from colleagues; and pandemic impacts on regular services and supports (Booth & Venville, 2020).

The pandemic has exposed or highlighted significant social inequities (Abrams & Detlaff, 2020), including favoring individuals with ready access to technology. Concern abounds regarding the impacts of the pandemic on already vulnerable populations (Amadasun, 2 020; Banks et al., 2020), such as people with disabilities (Farkas & Romaniuk, 2020), those living in poverty (Farkas & Romaniuk, 2020), racial minorities (Chowkwanyun & Reed, 2020), and people reliant on supports and services that have been subsequently reduced or become unavailable in the pandemic (Ross et al., 2021). Stereotypes and stigma against certain groups have arisen, with one example being stigma toward individuals of Asian descent (Farkas & Romaniuk, 2020). Additionally, there are reports that some services have been overwhelmed, while others have lacked clients and workflow (Abrams & Detlaff, 2020). Furthermore, many individuals, including social workers, have lost their jobs, contributing to economic strain among the profession (Farkas & Romaniuk, 2020).

Although the adaptability of the social work profession has been noted as a point of pride by some in response to the COVID-19 pandemic (Wiener et al., 2021), contradicting, shifting, and/or a lack of sufficient COVID-19 communication has been identified as frustrating the ability of social workers to meet the needs of their clients (Ross et al., 2021). Noted in the literature is the tenuous balance of attending to the needs of clients, yet also adhering to the protective measures of COVID-19; for example, seeking to ensure that family members have a chance to say goodbye to a loved one before or at end-of-life (Fox et al., 2021; Wiener et al., 2021), or the need for increased communication with medical staff to ensure that patients and family members have access to required resources (Ross et al., 2021).

Human resource (HR) pressures have been substantial during the pandemic. According to Adam et al. (2021), the safety of healthcare workers and their families must be prioritized by organizational leadership, in addition to providing enhanced mental health supports and resources to augment employee wellness amid the intense stress generated by the crisis. Absenteeism is an important concern in a pandemic, particularly within the healthcare context, which worsens as the pandemic deepens and its duration is extended (Yonge et al., 2010). Increased job scope and pressures place social workers in a tenuous position between their clients’ and their own needs and well-being.

Amid these challenges and uncertainties confronting social workers in the pandemic, the literature alludes to hope that the pandemic has heightened or will heighten opportunities for social workers to expand practice, increase interdisciplinary colleagues’ awareness of the psychosocial needs of patients and families, and amplify social work perspectives in policy and resource development (Ross et al., 2021; Wilson et al., 2021). However, to date, there is a dearth of literature that addresses pandemic-related experiences of social workers and/or impacts on social work as a discipline, and how social workers may be uniquely positioned to inform clinical and policy responses. To address these gaps, this study elicited the impact of the COVID-19 pandemic on healthcare-based social workers. Research questions were: (1) How has social work in health care been impacted by the COVID-19 pandemic?, (2) What are the experiences of social workers during the pandemic?, and (3) What recommendations do social workers offer to improve pandemic response in health care?

## Methods

Focus groups were convened, based in an Interpretive Description orientation (Thorne, 2016). As a qualitative approach, Interpretive Description elicits *on the ground* understanding and recommendations for practice and policy. Focus groups were facilitated by a semistructured guide in which open-ended questions addressed research aims. Focus group questions were as follows: (1) Can you describe your experiences practicing during the COVID-19 pandemic?, (2) What have been the impacts of the pandemic on your experience and morale as a social worker?, (3) How has the pandemic had a bearing on patients and families?, (4) What has been helpful to your practice during the pandemic, and what has made it more challenging than it could otherwise have been?, and (5) What is social work's contribution in a pandemic environment?. The focus groups were facilitated by DN, PS, and LH.

Group content was digitally recorded and subjected to verbatim transcription, line-by-line coding, categorization, and theme generation (Patton, 2014) supported by NVivo 11 qualitative data management and analysis software (QSR International, 2018). Data were reviewed, coded, and analyzed by two independent research assistants who were trained and supervised by DN who brings extensive experience in qualitative data coding and analysis.

Trustworthiness was demonstrated through a review of emerging findings by a subsample of *n*  =  6 participants via member checking, and subsequent peer debriefing with social work in health care clinicians and researchers. Both approaches indicated resonance of results with identified experiences and perspectives. Extensive referential adequacy reflects corroborating text quotes to illustrate reported themes, and memo writing and reflexivity were conducted throughout the research process.

### Sampling and recruitment

The sample comprised hospital-based social workers currently employed in hospitals and senior care facilities in western Canada, a large and diverse geographic region that includes both urban and rural regions, although participants generally worked in urban facilities. Managers informed social workers about the study and invited them to participate. Prior to participant involvement, information about the aims and processes of the study was shared with potential participants. They were informed that participant identity would remain confidential and any identifying information would be removed from transcripts. Informed consent was provided prior to study engagement, and the study was reviewed and approved by the University of Calgary Conjoint Faculties Research Ethics Board (REB18-1172).

Twelve focus groups were convened, comprising an average of five individuals per group. Two groups met in person within facilities (with required physical distancing and PPE), and the remaining 10 groups were held via videoconferencing technology due to safety protocols as the pandemic unfolded. Focus groups were held from June 2020 to March 2021, with a total of 67 BSW and MSW-trained social workers. Areas of social work practice are outlined in Table 1, and participants’ years of experience in social work in health care ranged from 1.5 to 40 years (Mean  =  11.5, Median  =  11). Participants worked in large urban cities (>1 million population) and small cities (50,000–100,000 population), with all regions having been profoundly impacted by the COVID-19 pandemic.

**Table 1.** Participant areas of social work in health care practice.

Participant service area	Participants
Medical/Surgical Care	17
Clinical Leadership	15
Rehabilitation	13
Geriatrics/Extended Care	12
Mental Health	10
Pediatrics	7
Oncology	4
Home Care/Community Care	2
Cardiology	2
Emergency Medicine	1
Critical Care	1
Palliative Care	1

*Note.* Some social workers covered more than one health service/area of practice.

## Results

The data reflected broad areas of experience and impact. Themes entailed: (1) practice shifts; (2) increased work and changing roles; (3) imposed restrictions; (4) problems in communication and decision-making; (5) distress, fear, and demoralization; and (6) *silver linings* amid challenges. Each theme is described below, along with corroborative text quotes.

### Practice shifts

Social workers described pivoting their practice to patient and system needs as dictated by the COVID-19 pandemic and practice directives. Delivery of care was shifted in volume and content, with the provision of services in other units by some social workers to offset staff shortages, and more services offered over the telephone or online which imposed challenges in terms of tangible issues such as form completion. Participants described decreased services during the pandemic, both within and out of the hospital, including patient referral targets. Some described rapidly pivoting to greater reliance on technology in practice delivery, which was deemed suitable for some, but not for others. For example, basic services that typically demanded face-to-face engagement, such as biopsychosocial assessments, were offered by some via technology, yet conducting these assessments online reportedly was made more challenging with the two-dimensional limitations of technology.

Shifting and demanding workloads were commonly noted, as illustrated by a participant who stated:

That's a challenge, getting your stuff done in the day. In addition, you’re… having to check your 101 emails in the morning. All of a sudden, you’re assigned to a meeting that you have to go to. There's mention of rollbacks with jobs… but you don’t have time to think about that, that's back here somewhere because you have to focus on your tasks today, whatever they be. You may have a plan when you first come in, but I’ll tell you by the time 3:30 comes around, it's a totally different thing. And then it's back here again. [Participant A3]

The demanding nature of social work in the pandemic was described to entail frequent shifts. A participant stated:

I’ve learned four different jobs since COVID-19 started. I had to learn how to screen people at the door. I was sent to a medicine unit to learn that, and then over to another unit. I just felt like I can’t keep all this information, like I’m just full. [Participant B1]

Several participants described personal and professional struggles due to tensions when asked to work outside their scope of practice. A participant stated that re-assignments sometimes “caused a level of anxiety” [Participant B2], and participants variably conveyed being overwhelmed in their daily work.

Participants described variation in practice, including the location of work—either in hospital or at home. Working at home presented adjustment and difficulty; for instance, some social workers lacked privacy to have confidential conversations, particularly if their home workspace was not conducive to such work activities. For others, coming to hospital given concern over the perceived risk of COVID-19 exposure, was troubling. Some struggled over the lack of choice to engage in work in a way that was deemed safe. For individuals working from home, several described relief, and yet a sense of guilt in not being exposed to the level of risk faced by their colleagues in hospital or long-term care settings.

#### Increased work and changing roles

Social workers were periodically asked to be the “bearers of bad news” in conveying visitation restrictions or other pandemic-related regulations to patients and families. They described moral distress due to being placed in a conflictual role relative to their values and the traditional social work role of seeking quality of life and accessible care. Commitment to care and navigational assistance to patients and families was variably supplanted by additional roles of enforcing pandemic-related restrictions. Service provision was further hampered by social distancing restrictions, including barriers reflective of PPE. In thinking about these requirements and practice shifts, a social worker described:

The lack of ability to connect the way that we usually do with patients is really hard because I’m going to see patients, and I want them to know that I’m an ally, and you smile, and you use yourself. But you can’t be close to them, you can’t express yourself in ways that you know make them feel comfortable, and that they can trust you, so that's just a whole other piece of it—that physical nature of social work is just being able to go over and put your hand on their arm and you can’t even do that…. That's been challenging. I try to smile with eyes but that doesn’t always work. [Participant B3]

### Imposed restrictions

Imposed restrictions were viewed to disproportionately affect populations largely served by social work, and in particular, individuals with social determinants of health barriers—many of whom were noted to have been more severely impacted by the service ruptures associated with the pandemic such as individuals with less resources or limited access to online technology, those from remote regions, elderly people, and non-English speakers. A social worker shared:

When patients and families do not speak English, a Zoom call is not adequate. I don’t have any way of communicating with them, so I would either need to do that in person using an interpreter through the language line or on the phone. And while I have come leaps and bounds with being able to connect with people virtually and seeing their faces, I don’t feel I’m able to do that on the phone in the same way, not for a two-hour assessment. So, there are some instances when language is involved that virtual care does not work. [Participant C2]

In another instance of imposed restrictions, participants described a lack of PPE early in the pandemic, and a designation of essential services that, in some cases, did not include social work; hence, access to PPE early in the pandemic was restricted particularly when in short supply. This left some social workers feeling at heightened risk of contracting COVID-19.

Some participants were assigned to roles of conveying care and system restrictions (e.g., visiting rules, public health, or institutional/unit directives) to patients and families which often resulted in patient and family struggle, and left social workers deeply concerned about immediate and longer-term deleterious impacts. In some instances, participants reported pushing back against restrictions and guidelines, as possible:

I had a patient recently who actually had a panic attack in my office, and I have the space in my office to create more than 6 feet and in order to help ground her… I pulled my mask off because she had taken her mask off, and she said, ‘I need to see your face’, and so we did that for about 60 seconds. [Participant C2]

Several participants reported personal struggle over the requirement to uphold pandemic rules and care guidelines such as enforcing the separation of families at particularly challenging times such as health crises or the end of life. One social worker described:

Being in the ED yesterday and speaking to this [elderly] gentleman whose wife is in the trauma bay, and he can’t go back there and he's distraught and just wants to make sure she's okay, and I can’t give any real information about how she's doing because that will come from the physician. But also letting him know that he cannot stay here in the waiting room. And so it does put you in this really vulnerable, raw place… want[ing] to help them and you feel helpless in [not] being able to help them, be there for them, and support them. [Participant C4]

Another participant added:

Dealing with the visitor policy… has been very, very stressful and caused a lot of grief to us as social workers and to staff. Especially with those end-of-life situations where we’re trying to… plead for family members to come in to say good-bye…. Everybody seems to have their own opinions… on how to deal with that, and that has caused some tension on my team. [Participant D6]

### Problems in communication and decision-making

Although communication was viewed as central to pandemic management, decision-making and the communication of care-related decisions were viewed by some to periodically lack receptivity to the range of needs of patients and their families, as well as clinicians and their practice *on the ground*. Participants described instances in which decisions related to family visitation in a unit or facility were perceived to be incommensurate with the extent of what was needed or appropriate in that context. Some participants reported concern about public health guidelines that were implemented with challenge and impact on patients and teams. As an example, a participant described a conundrum in care imposed by care guidelines relative to a homeless patient:

It's like someone who maybe has no fixed address, and (we’re) trying to… connect them to the right resources to maybe move towards housing in the future. …There's this push to get people out of acute care because we want to empty beds especially if they’re in a COVID unit…. It's like, ‘Well let's get people with no fixed address over to the [local isolation hotel for the homeless population] for them to isolate.’ But that takes away any time that I would have connecting them to resources. [It's] just kind of hoping that someone at [the hotel] will have time to do this and I am left wondering, ‘am I doing justice to this patient?’. I don’t know that I’m fully doing my job to its full potential right now, and this person might just wind back up in acute care for the same problem. [Participant D1]

Concerns over frequently changing pandemic directives were exemplified by a participant who stated, “by the time [communication] gets down to the ground, it's like the telephone game. I think some[thing] gets lost in that passing on because… there's lots of inconsistencies between information.” [Participant E1]

External messaging in the media was viewed by some to be upsetting and demoralizing, as exemplified by news stories about citizens in the broader community who denounced the virus and its impact. Some participants described distress due to putting themselves and their loved ones at risk by coming into the hospital daily while community members discounted the extent of the pandemic, further risking virus spread by not observing precautions. For some participants, these messages were a painful juxtaposition of their heightened risk and struggle in the face of community and societal ruptures.

### Distress, fear, and demoralization

Many social workers described deep strain and grief: “I had to go to units… and [provide] support, and I saw entire units, you know, [large numbers of] people die within a short period of time, you know, we’re talking three, four weeks. That's pretty tough.” [Participant F1] Amid distress at work, participants conveyed vulnerability and fear by virtue of experiencing the pandemic both at work *and* at home, that is, professionally and personally, and being uncertain about virus contagion and risk to themselves and their family, particularly due to continued attendance at the hospital. As a service provider, they described coming to work and solving problems rapidly and professionally, supporting the team, maintaining a positive outlook, and thus, masking personal worries and negative emotions. Several described “camouflaging” their sense of vulnerability, fear, and frustration due to seeking to be professional in addressing the pressing challenges of the workday and context.

Work-related struggles were described to extend to home as participants conveyed extensive worry and extreme safety precautions; examples included showering and changing clothes after hospital shifts, relocating to a separate home location to ensure physical distancing from family, and largely avoiding contact with others at home and in the community, particularly early in the pandemic, in order to minimize infection spread risk. Such responses shifted with the progression of the pandemic relative to knowledge about infection spread and pandemic severity and intensity at given points in time. Several identified the contradictory experiences of receiving praise from community members for their service in health care, yet stigma due to working in a hospital.

### Silver linings

Along with the predominance of strain in the pandemic, participants described elements of learning and adaptation that have emerged in the pandemic. Participants noted markedly increased technology use in providing care, compared to prior to the pandemic. Several noted resultant gains in service access and convenience for some patients and families. Technology use was seen as beneficial for individuals who live substantial distances from treatment centers, and/or have easy access to, and are comfortable with, online communication.

To conduct online-mediated work in the pandemic, some social workers received new or updated hardware and software for pivoting to online-mediated care. Many believed that heightened technology use in service delivery, as required in the pandemic, would likely continue, at least to some degree, after the pandemic. Yet caution was raised in that this modality, while beneficial for some, was thought to not be optimal for certain populations (e.g., some seniors with less computer experience or acumen).

Some participants in supervisory or clinical leadership roles described better processes for the escalation of concerns through the health system due to increased receptivity to redressing pressing care challenges associated with the pandemic and care needs. Examples were offered in which interdisciplinary teams had demonstrated a heightened understanding and appreciation for the integral role of social work in assisting patients and families as well as supporting team members.

Participants consistently described the importance of collegial support in the pandemic. Exemplifying the importance of such support, a participant stated:

There are days where I’ll touch base and say ‘I’m losing my mind.’ And, somebody else says ‘so am I.’ Okay, I’m good. That's validated that I’m not alone. It may sound silly, but essentially… it's knowing that you’re not in this alone, that you can talk to someone not necessarily to have them solve your situation, but just…know[ing] they’re going through the same thing and information sharing. If we [come] across something, we need to make sure we fan that out so that the next person is not having to jump hurdles. [Participant B4]

As illustrated above, many participants described drawing on and appreciating intra- and interdisciplinary support. Yet others felt more isolated, with some participants suggesting that teams that were less supportive prior to the pandemic, often remained so during the pandemic—while supportive teams rallied to offset individual isolation and strain often via technology application and connection.

## Discussion

Given substantial practice and experiential challenges, social workers described fatigue and varying levels of struggle at the onset of the pandemic and as it persisted over time. For several, they described fewer margins in their workday as a result of demands and stresses. Many experienced distress from observing patients and families suffering. Participants described numerous instances in which previously held practices were supplanted by shifts in care which over time, resulted in fatigue, and in some cases, demoralization.

The pandemic has amplified systemic gaps, with substantial personal and professional impacts. Of this impact, Farkas and Romaniuk (2020) argue, “we will face continuing problems of job loss, economic hardship, mental and physical strains among all levels of society after the threat of infection and illness has passed” (p. 79). Social workers in Ross et al.'s (2021) study likewise predicted a “second pandemic”, which likely will disproportionately affect the most vulnerable members of the population due to the current and continuing impact of the COVID-19 pandemic on economic conditions, population mental health, and service access (p. 14). Ebor et al. (2020) note that social workers have a duty to address these intersecting vulnerabilities. We concur, while recognizing and applauding social workers for addressing, with others, systemic and societal inequities, and advocating for equitable care to vulnerable communities. Yet, we also identify concern over professional strain imposed by these pandemic circumstances.

### Shifting experience over the course of the pandemic

**Figure 1.**
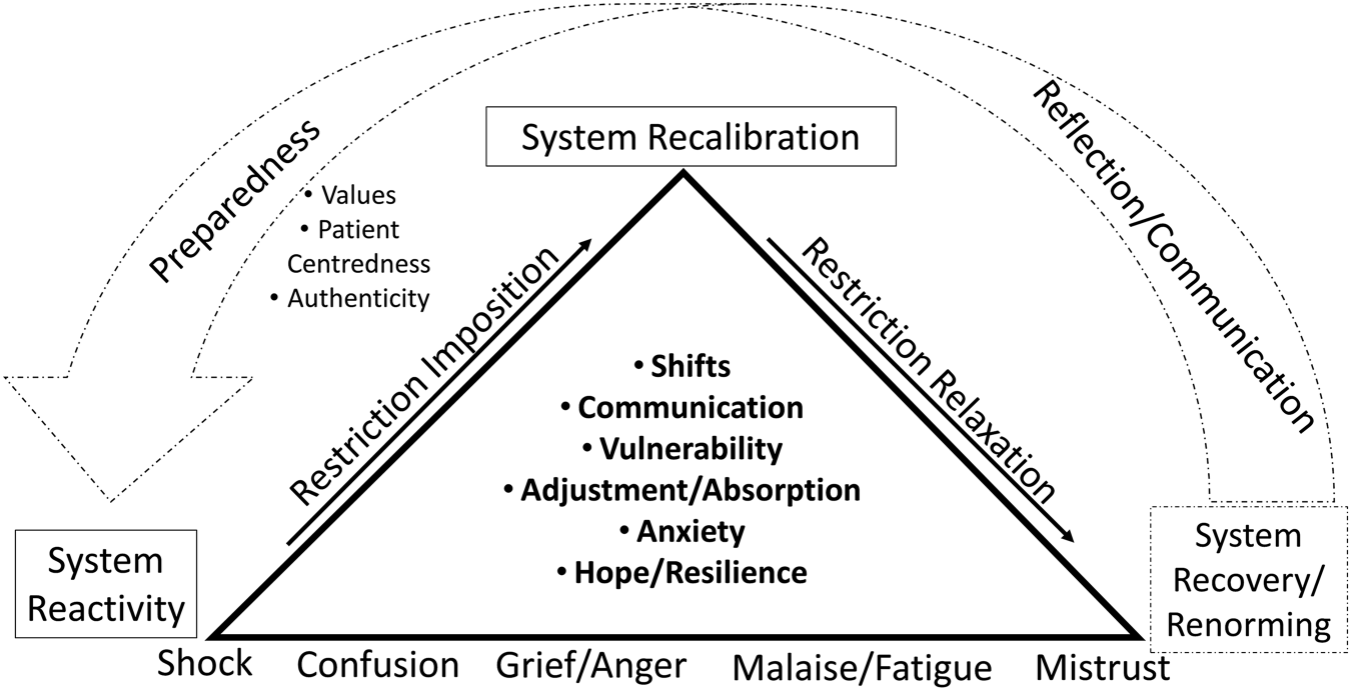
Emerging processes over time.

Social workers’ experiences of the pandemic were notably marked by adjustment as well as shifting impacts over time, as depicted in Figure 1. Upon the pandemic's onset, the system reacted with rapid community and service shifts. For social workers, this was described personally and professionally as a time of adjustment in their practice, resulting in confusion and uncertainty about risk and vulnerability. They conveyed their experiences in terms of seeking to calibrate and find their moorings in practice despite fear and anxiety related to the requisite of pandemic and care shifts. Over time, these challenges were amplified by grief and struggle, and in some cases, frustration over the ruptures in services and extended nature of the pandemic. Systemic decisions sometimes were viewed to impose a difficult tension of public health protection and health system management versus individual patient/family well-being, resulting in heightened distress for social workers in terms of service delivery conundrums, conflict with values, and strained personal/professional morale.

Over the deepened and extended duration of the pandemic, participants described fatigue and, in some cases, resentment about the continued shifts in policy, and inconsistency or lack of justification for policy decisions relative to the difficult balance of person-centeredness versus population safety in the context of patient care. As social workers walked through these experiences and emotions over time, common elements emerged such as frustration and compassion fatigue, yet deep contribution to patients/families and teams.

Considering the potential of future pandemics, it is important to learn from this COVID-19 pandemic experience for proactive planning that reflects deeply held values and consideration of priorities such as person-centered care, frontline service issues and realities, and downstream impacts on patients, families, and the healthcare workforce. HR and operational priorities such as workforce support and decision-making transparency are paramount, as is consistent and targeted communication.

The extended duration of the pandemic (over two years to date) invites systemic attention to pressing and potentially shifting health workforce needs over the course of the pandemic as well as after the pandemic. Considering the needs of health care providers and various disciplines as they move through (and past) a pandemic requires tailored HR responses, premised on listening to the needs of these stakeholders and offering support such as mechanisms to respond to front-line care needs, potential grief and trauma counseling, and other relevant resources, as indicated.

Relevant and up-to-date pandemic information emerged as integral to a strong pandemic response in: (i) ensuring sufficient yet targeted information, and (ii) helping to manage the barrage of pandemic messaging in the media and elsewhere. Transparency is needed in rapidly disseminating and justifying the reasons for healthcare system decisions as they relate to patient care and service planning.

Building an evidence-informed base of pandemic-related practices and processes in preparation for, during and after pandemic surge, invites attention to *at risk* populations reflective of Social Determinants of Health barriers and amplified attention to equity, diversity, and inclusion considerations as well as the workforce serving these populations. This invites proactive pandemic planning that balances the tenuous priorities of public health *and* person-centered care. Applying an integrated ethical lens to understanding need and providing commensurate services is an ongoing challenge amid public health demands. The development of coordinated and responsive systems of care can be supported by consultation with frontline personnel in order to nurture optimal workflow and support frontline clinical care needs in the constrained context of the pandemic. Further, managing and supporting work/life/health balance will be integral in post-pandemic recovery.

The difficult and prolonged experience of the pandemic has amplified our global awareness of system gaps and inequities, as well as shone a light on the integral role of social workers as they have: (i) addressed challenges that marginalized populations have faced, (ii) contributed to person-centered health care, and (iii) supported intra-and interdisciplinary colleagues. Commensurate with this integral contribution to society and health care, it is important to consider the amplified need for social work in health care, particularly given potential surge in post-pandemic mental health challenges in the community, and the likely possibility of future geo/health/environmental crises. In this endeavor, social work's commitment to, and acumen in, socially just care particularly to those with heightened vulnerability and marginalization, invite an active role for social work in contemporary health care including in proactive pandemic planning and recovery.

## Limitations and Research Implications

As in all studies, this research has limitations. Given its exploratory nature, the study relied on experiential and perspectival data from qualitative focus groups. Interviews may have offered more depth regarding personal impacts, particularly if a given social worker may have felt less safe to honestly convey diverse or difficult experiences or feelings in a group. Although we offered an interview option, all social workers opted to participate in a focus group. The focus groups offered an efficient and conversant approach to elicit experiences and perspectives, yet an inherent risk, in turn, entailed possible “groupthink” which may have swayed or discouraged minority perspectives. On the other hand, we encouraged openness in expressing a wide range of perspectives, and the use of focus groups allowed for a collective conversation that was reported by many participants to be cathartic and supportive. Although not the aim of a focus group, support for social workers in such a difficult time as the pandemic was seen as an important vicarious benefit.

Although necessary due to physical distancing restrictions, our reliance on technology to collect data introduced a limitation of potentially being less sensitive to the nuances of nonverbal expression and observation. On the other hand, the intensity of identified experiences offered in focus groups suggests that participants generally expressed their perspectives within this modality of data collection. Finally, while advantageous to collect data over a lengthy period of time during a pandemic, that is, June 2020 to March 2021, we acknowledge that the experiences of social workers over the duration of the pandemic indeed shifted, thus introducing heterogeneity of context over the course of time and pandemic conditions. Conversely, we only collected data over a portion of the pandemic (although an extended timeframe). Nuanced shifts in experiences from initial/early to mid to late phases of the pandemic thus may not have been captured to the extent experienced by social workers. Although the relatively extended time of data collection over the duration of the pandemic may reflect limitation *and* benefit, it nonetheless demarcates a time stamp of social work experience and contribution at this geo-health juncture in history.

It is recognized that these data were collected from a subset of individuals who were willing to participate in the study and may not represent the experiences of all social workers across the health care system, and particularly not necessarily of those in different jurisdictions or health care systems. Accordingly, a larger and representative sample is recommended in future study. Additional healthcare providers’ perspectives would offer interdisciplinary texture for a broader understanding of team members’ roles and experiences.

Further study about the challenges and intersecting layers of administrative decision-making in pandemics is invited, as is exploration about the experiences and processes of care from the perspective of patients and their families. Research is also needed to examine shifts beyond the pandemic, along with what supports/services mitigate negative impacts and foster resilience. Longitudinal study of post-pandemic experiences and responses over time, among the range of the various stakeholders, is recommended. Such inquiry may offer insight into education, support, policy development, and pandemic planning.

Finally, research with robust methods is needed in testing differential impacts of a pandemic on various disciplines including social work, as well as on subpopulations such as clinical/patient groups, socioeconomic or ethnocultural-based communities, urban versus rural populations, and geographic regions. Intervention-based study is warranted in building evidence-informed strategies and supports to limit negative impacts and conversely nurture recovery.

## Conclusion

The COVID-19 pandemic has monumentally affected the global community and more specifically, health care delivery, including the provision of social work and the experiences of social workers. This study has amplified these impacts and reminds us of the important contribution of social workers in proactive care, advocacy, and intra and interdisciplinary support during a pandemic.
